# The effects of extraction techniques and quantitative determination of oxalates in *Nerium oleander* and feeds

**DOI:** 10.4102/ojvr.v86i1.1611

**Published:** 2019-05-29

**Authors:** Kedibone G. Kgosana

**Affiliations:** 1 Toxicology and Ethno-veterinary Medicine, Agricultural Research Council, Onderstepoort, South Africa

**Keywords:** antinutritive factors, extraction, decoction, infusion, feed, toxins

## Abstract

Shrubs represent the most affordable and accessible form of feed that livestock can rely on to acquire both essential and non-essential elements of life. In addition to their inherent toxins, they contain endogenous substances commonly referred to as ‘antinutritive factors’ (ANFs) that often interfere with the utilisation of nutrients. Their abundance may lead to severe clinical trauma. Hence, the objective of the study was to investigate the effects of different extraction techniques on *Nerium oleander* L. and animal feeds as well as to quantify oxalates. Organic (hexane, acetone and methanol) sequential and aqueous (infusion and decoction) extractions were explored. Qualitative and quantitative analyses were conducted to determine the presence of various phytochemicals and oxalate contents as putative ANFs, respectively. The results showed higher extraction yields of 22.6% and 43.1% in the decoction and infusion of *N. oleander*, respectively. The quantification methods were validated for linearity, accuracy and precision. Oxalate contents of 6.76 ± 0.245 (0.65%) mg/g and 5.74 ± 0.236 mg/g dry weight (0.55%) were obtained in *N. oleander* and feeds, respectively. This difference was statistically significant with *p* < 0.05. Percentage recoveries of 98.5 (percent relative standard deviation [% RSD] = 2.3), 85.7 (% RSD = 1.03) and 80.3 (% RSD = 1.22) at 76%, 95% and 112% fortifications were obtained, respectively. Relative standard deviation for precision was 0.99% and 1.13% at 0.33 mg and 0.39 mg fortifications, respectively, while reproducibility showed 2.21% RSD. Therefore, these methods can be used to provide a valuable basis for qualitative determination of ANFs, particularly in shrub foliage.

## Introduction

Antinutritive factors (ANFs) occur as natural plant and animal feed constituents generated by the normal metabolism of species or as a result of inactivation of some nutrients or metabolic utilisation of feed (Kumar 1991). They may also occur as artificial factors added during processing or as contaminants because of fertilisers, pesticides, industrial pollutants and heavy metals in the ecosystem (Soetan & Oyewole 2009). Their action is essentially to interfere with nutrient utilisation, thereby deteriorating animal health (Maheswari et al. 2010). Antinutritive factors found in plants include, amongst others, saponins (triterpenes), tannins, trypsin inhibitors, oxalates, cardiac glycosides, alkaloids, phytates, haemagglutinins (lectins), cyanogenic glycosides, glucosinolates, S-methyl cysteine sulphoxide and gossypol (Fletcher et al. 2010; Kiranmayi 2014).

Endogenous ANFs such as oxalates can be generated from metabolic activities of ascorbates, amino acids and glycolates (Williams & Smith 1983). However, the toxic nature of oxalates is of agricultural importance because they occur in plant tissues as soluble and insoluble salts (Liu et al. 2015). The soluble and insoluble salts form as a result of bonding between oxalic acid with alkali metals (Na, K) and alkaline earth metals (Ca, Mg), respectively. The insoluble oxalate complexes have an adverse effect on mineral bioavailability as they prevent absorption of minerals (Adeniyi, Orjiekwe & Ehiagbonare 2009; Morrison & Savage 2003). Moreover, high rates of endogenous oxalate synthesis and intestinal oxalate absorption may lead to a condition known as ‘hyperoxaluria’, a primary risk factor in the formation of oxalate-containing kidney stones (Holmes, Goodman & Assimos 1995). Previous studies also reported acute toxicity and death in cattle that consumed pastures containing 6.9% of oxalates as a result of hypocalcaemia and chronic nephrosis, respectively (Seawright, Groenendyk & Silva 1970). In contrast, low oxalate intake in horses was associated with metabolic bone diseases (Walthall & McKenzie 1976) because minerals play a significant role in the skeleton. Generally, high oxalate contents are found in leaves, while lower contents are in the seeds and stems; hence reasonable strategies to quantify, eliminate and reduce these molecules in animal feeds and foliage are urgently required.

Previous studies and reviews showed the strengths and limitations of these molecules and their removal from animal feeds using traditional techniques such as maceration, infusion, percolation and other state-of-the-art techniques including thermal processing, fermentation, bioprocessing, microwave-assisted, ultrasound-assisted or sonication, accelerated-solvent and supercritical fluid extractions (Azwanida 2015; Chen et al. 2013; Ibrahim et al. 2002; Luo & Xie 2013). However, the high costs associated with the removal of these undesirable molecules often result in the production of reduced feed quality, which ultimately results in the declined progress of animal health advancement. In addition, many farmers are prompted to lead their livestock to graze in the open veld or rangelands, not realising the risks to livestock grazing directly on pastures and/or tree or shrub foliage. Poor management systems in the open veld are a leading cause of a growing number of life-threatening accidental exposures. Unfortunately, most exposures have not been properly addressed throughout developing countries, particularly on the African continent, with diverse flora. The risks are frequently inevitable and most research interventions across the globe are poised to find appropriate and effective mitigation strategies to benefit livestock.

Despite higher expenditures on processes involved in reducing ANFs and toxic factors in plants, other low-cost methods such as boiling, roasting, simmering and blanching have been employed effectively. In view of the antimineralising effect of water-soluble oxalates, contents of oxalic acids may be lowered by boiling the feed or forage and pouring away the water (Gontzea & Sutzescu 1968). Recently, some investigations with a predominant focus on similar methods revealed remarkable findings. For instance, boiling of *Moringa oleifera* leaves resulted in a significant reduction of oxalate, cyanide and trypsin inhibitor levels by 85.3%, 88.1% and 78.8%, respectively (Sallau et al. 2012). Previous investigation also showed a reduction of oxalate contents ranging between 2.62 mg/g and 2.76 mg/g versus 3.04 mg/g and 3.12 mg/g in boiled and raw samples of *Arachis hypogaea* varieties, respectively (Mada et al. 2012). This shows the need for development of many methods to complement the poor infrastructural settings in sub-Saharan Africa.

Hence, this study aimed to develop affordable methods that can be used by farmers and feed producers to eliminate various antinutritive and toxic factors in foliage. *Nerium oleander* (Apocynaceae) was selected based on the fact that perennial shrubs are always accessible for livestock browsing and that the shrub has been intensively investigated for cardiac glycoside poisoning (Calderón-Montaño et al. 2014; Te Riele et al. 2013).

## Research method and design

### Plant collection

Fresh leaves of *N. oleander* were collected during the month of May 2017 in the Soshanguve Gardens (25.5226° S, 28.1006° E) in Gauteng Province, South Africa. The voucher specimen was deposited in the H.G.W.J. Schweickerdt Herbarium of the University of Pretoria, and the University of Pretoria herbarium number 123 710.

### Materials

All analytical grade reagents and standards used in the study were purchased from Sigma-Aldrich (Johannesburg, South Africa) and Merck (Modderfontein, South Africa) while high-performance liquid chromatography (HPLC) grade solvents were purchased from Merck. A bag of AFGRI Animal Feed (bovine) (code R1153P) comprising crude protein, crude fibre, moisture, crude fat, calcium and phosphorus was obtained at the Agricultural Research Council stores and used as a negative control for qualitative and quantitative analysis.

### Plant and animal feed processing

The leaves that were collected were washed with tap water, fan dried at room temperature for 21 days and then ground into powder using a rotor mill, ZM 200 (Retshch GmbH). Similarly, animal feed (pellets) was ground into powder using a rotor mill.

### Animal feed and plant extractions

#### Organic sequential extractions

The powders from both animal feed and plant leaves were weighed separately in a ratio of 1:3 w/v into solvents in sterile bottles. The mixtures were shaken vigorously for 16 hours at room temperature on a benchtop shaker (Labotec, model no. 202, Midrand, South Africa) and filtered through Whatmann filter paper No. 50 (24.0 cm). The extraction process was repeated twice, the filtrates were combined and dried under reduced pressure before re-extraction with the next solvent. The sequence of the solvents was hexane, acetone and methanol.

#### Aqueous extractions

For the decoction, two replicates of each powdered material were measured. Amounts ranging between 50.20 g and 50.72 g of feed and plant powders were weighed separately into each beaker. Three hundred millilitres of warm water (55 °C – 65 °C) was added into each beaker. The mixtures were then boiled for 20 minutes at 80 °C – 100 °C, allowed to cool at room temperature, strained with a mutton cloth and the extracts were subsequently stored at -20 °C. Two replicates of each powdered material were also measured for the infusion. Amounts ranging between 50.07 g and 50.46 g of feed and plant powders were weighed separately into each beaker. Three hundred millilitres of warm water (55 °C – 65 °C) was added into each beaker. Then 10.0 g of sodium hydrogen carbonate and 5.0 g of citric acid were added. The mixtures were allowed to cool at room temperature; they were strained with a mutton cloth and the extracts subsequently stored at -20 °C. The frozen extracts were lyophilised at -45 °C using a VirTis benchtop SLC (SP Scientific) freeze dryer.

#### Qualitative analysis of feed and plant leaf extracts

The phytochemical screening procedures were carried out as previously reported (Ali 1998; Sofowora 1982; Tamokou, Mbaveng & Kuete 2017), with slight modifications. Three replicates per extract were carried out in all test methods. All test methods were carried out at ambient temperature unless otherwise stated, for example, boiling and cooling at room temperature or on ice.

#### Bornträger test

An extract was boiled with dilute sulphuric acid, filtered and the filtrate was extracted with chloroform. A few drops of ammonia were added to the organic phase to observe a pink to red colour because of the presence of anthraquinones.

#### Foam test

About 0.5 mL of the 100 mg/mL extract was dissolved in 19.5 mL distilled water in a centrifuge tube. The mixture was shaken vigorously for 15 min. A 2 cm foam layer indicated the presence of saponins.

#### Vanillin–hydrochloric acid test

One gram of vanillin was dissolved in 10 mL of methanol. A test reagent was prepared by adding 10 mL of concentrated hydrochloric acid. The extract was then treated by the addition of a few drops of vanillin–HCl reagent and a pink or red colour was observed because of the formation of vanillin–phloroglucinol condensates.

#### Gelatin test

Two millilitres of a 1% gelatin solution containing 10% sodium chloride was added into 5 mL of extract (20 mg/mL). White precipitates indicated the presence of phenolic compounds.

#### Xanthoproteic reaction

Two millilitres of extract (100 mg/mL) was warmed with nitric acid. Formation of yellow precipitates indicated nitration of an aromatic ring present in tyrosine, tryptophan and phenylalanine.

#### Ninhydrin test

Two to five drops of 1% ninhydrin (in acetone) solution were added to 2 mL of extract. The mixture was boiled for 1–2 min and the formation of a red to violet–purple colour indicated the presence of amino acids.

Solvent-free extract (50 mg) was stirred with a few drops of dilute hydrochloric acid. This was filtered and the filtrate collected to perform Mayer’s, Wagner’s and tannic acid tests.

#### Wagner’s test

A few drops of Wagner’s reagent (1.27 g of iodine and 2 g of potassium iodide in 100 mL distilled water) was added to the filtrate. A reddish-brown precipitate indicated the presence of alkaloids.

#### Mayer’s test

At least 1 mL of the filtrate was treated with two to three drops of Mayer’s reagent (1.358 g mercury chloride in 60 mL distilled water plus a solution of 5 g potassium iodide in 10 mL. The solution was made up to the volume of 100 mL). A pale yellow or creamy white precipitate indicated the presence of alkaloids.

#### Tannic acid test

About one to two drops of a 10% aqueous solution of tannic acid was added to 1 mL of the filtrate. An amorphous or crystalline precipitate confirmed the presence of the alkaloids.

#### Liebermann–Burchard’s test (cardiac glycosides)

About 500 mg of the dry extract was dissolved in 1 mL of acetic anhydride. The mixture was cooled on ice and a few drops of concentrated sulphuric acid was added. A change from violet to a blue-green colour indicated the presence of steroidal nucleus (aglycone of the cardiac glycosides).

#### Liebermann–Burchard’s test (sterols)

The plant extract (100 mg) was dissolved in 1 mL chloroform and filtered. The filtrate was treated with acetic anhydride, boiled for 2 min and allowed to cool. A few drops of concentrated sulphuric acid were slowly added and the formation of a brown ring at the junction indicated the presence of sterols.

#### Salkowski test (cardiac glycosides)

About 500 mg of the dry extract was dissolved in 2 mL chloroform. A few drops of concentrated sulphuric acid were added. A reddish-brown colour at the interface indicated the presence of a steroidal ring (aglycone portion of cardiac glycosides).

#### Salkowski test (terpenoids)

The plant extract (100 mg) was dissolved in 1 mL chloroform and filtered. The filtrate was treated with a few drops of concentrated sulphuric acid, shaken and allowed to stand for a few minutes. Formation of a yellow colour at the lower layer indicated the presence of terpenoids.

#### Alkaline reagent test

Two millilitres of the extract (100 mg/mL) was treated with a few drops of 20% sodium hydroxide solution. A formation of an intense yellow colour, which became colourless on addition of dilute hydrochloric acid, indicated the presence of flavonoids.

#### Oxalate test

Two millilitres of the extract was treated with a few drops of glacial acetic and a dark green colouration indicated the presence of oxalates.

### Quantitative analysis of feed and plant leaves

#### Instrumentation, chromatographic conditions and sample preparation

The purpose of this analysis was to quantify oxalates present in plant leaves and animal feed. High-performance liquid chromatography analysis was performed on an Agilent 1260 Infinity Quaternary LC (Agilent Technologies) and Agilent 1100 Series (Agilent Technologies) based at two laboratories (Toxicology and Residue) for reproducibility purposes. The HPLC instrument from each laboratory comprised a binary pump, high-performance degasser, high-performance auto-sampler, column thermostat and a variable wavelength detector. The analytical column used was an Eclipse XDB-C18 4.6 mm ID × 250 mm (5 *µ*m) 80Å. The chromatographic condition consisted of 20 mM sulphuric acid mobile phase, 1 mL/min flow rate for 10 min at a wavelength detection of 210 nm, ambient temperature and injection volume of 50 *µ*L. The sample was prepared using previously reported methods (Ruan et al. 2013). A 1 mg/mL stock solution of sodium oxalate was prepared and appropriate volumes from the stock solution were further diluted to prepare standards of varying concentrations.

#### Method validation

The quantification method was validated for linearity, accuracy, precision, limit of detection (LOD) and limit of quantitation (LOQ).

**Linearity:** Ten different concentrations ranging from 10 *µ*g/mL to 100 *µ*g/mL were prepared from 1 mg/mL stock solution of sodium oxalate. About 50 *µ*L of each solution was injected into the HPLC using an auto-sampler and three replicates were carried out. The calibration curve was created by plotting the average peak areas against the concentrations. The calibration curve was used to evaluate linearity of the method by calculating the coefficient of correlation, slope and intercept values.

**Accuracy and recovery:** Recoveries of sodium oxalates were calculated to determine the accuracy of the method. A known concentration of standard solutions (76%, 95% and 112%) was added to the analysed sample solutions and the injections were done in triplicates. The calibration curve was used to estimate the amount of the standard.

**Precision:** This study assessed precision with respect to intra-day, repeatability and reproducibility. The intra-day precision was performed in one day. To evaluate intermediate precision, the HPLC Agilent 1260 and Agilent 1100 instruments were used on different days to analyse replicated injections of the sample solutions. Conversely, four replicates of powder samples were prepared and determined simultaneously in order to evaluate repeatability. Reproducibility was evaluated by expressing precision between the two laboratories.

**Limit of detection and limit of quantitation:** The LOD and LOQ were determined by *k*σ/S, where *k* is constant (3.3 for LOD and 10.0 for LOQ), σ is the standard deviation of the sample response (blank) and S is the slope of the calibration curve.

### Data analysis

An online Mann–Whitney U statistical tool was used to compare differences between the feed and plant leaf samples. The threshold was set to 0.05 and the mean values were calculated and compared in order to determine the *p*-value.

### Ethical considerations

This article does not contain any materials that violate any personal or proprietary rights of any other person or entity.

## Results

**FIGURE 1:**
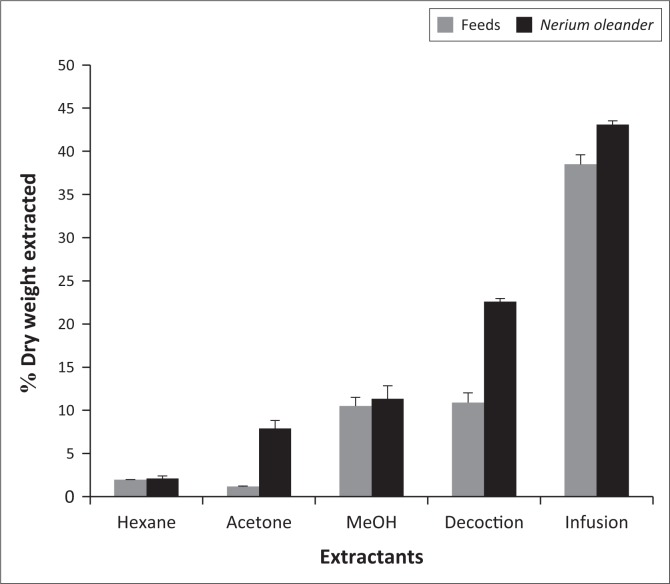
Efficiencies of organic sequential and aqueous solvent extraction systems in animal feed and *Nerium oleander*.

Extraction efficiencies of phytochemical constituents that may pose a serious health problem as ANFs and toxic factors in plants were evaluated using two solvent systems: organic sequential and aqueous extractions. At least three organic solvents – hexane, acetone and methanol – with different polarities were explored while for aqueous extraction, the decoction and infusion were prepared. Even though the animal feed showed lower extraction yields when compared to *N. oleander*, a well-defined ascending yield trend with increasing solvent polarities was obtained (Figure 1).

For organic sequential extraction, hexane efficiency was almost the same in *N. oleander* and the animal feed as it gave yields of 20.99 ± 0.743 g/kg dry weight (DW) and 19.61 ± 0.182 g/kg DW, respectively (Figure 1). In contrast, acetone resulted in higher extraction yields in *N. oleander* in comparison to the animal feed. Similarly, methanol showed almost the same extraction efficiencies in both samples. Furthermore, approximately a twofold and onefold increase in extraction yields in *N. oleander* compared to the animal feed was obtained in the decoction and infusion, respectively.

Qualitative analysis was carried out in order to evaluate safety, reliability and efficiency of the extracts derived from two extraction techniques. Table 1 shows a summary of various phytochemical constituents found present in both the animal feed and *N. oleander*. Interestingly, no traces of sterols, saponin glycosides or tannins were found in any of the extracts from the animal feed. Similarly, no traces of xanthoproteins were found to be present in the *N. oleander* extracts.

**TABLE 1:** Qualitative analysis of selected phytochemicals in feeds and *Nerium oleander*.

Phytochemical tested	Test	*Nerium oleander* extracts					Feed extracts				
		A	H	M	D	I	A	H	M	D	I
Terpenoids	Salkowski	-	-	-	-	+++	-	-	-	+	+
Cardiac glycosides	Liebermann–Burchard	+	+++	++	-	-	+	-	-	-	-
	Salkowski	-	-	-	+	+++	-	-	+	+	+
Saponin glycosides	Foam	-	-	+++	+	+++	-	-	-	-	-
Sterols	Liebermann–Burchard	++	-	-	-	-	-	-	-	-	-
Phenols	Gelatin	-	-	-	-	+++	-	-	-	++	++
Flavonoids	Alkaline	-	+++	-	+++	-	-	++	++	++	++
Tannins	Vanillin–HCl	-	-	-	++	-	-	-	-	-	-
Alkaloids	Wagner	+++	++	+++	++	++	+	+	++	-	++
	Tannic acid	+++	+++	+++	+++	+++	+++	+++	+++	+++	+++
	Mayer	++	++	++	+	++	+++	+++	+++	+	++
Anthraquinone	Bornträger	++	-	-	++	++	++	-	-	-	-
Proteins	Xanthoproteic	-	-	-	-	-	-	-	-	++	++
Amino acids	Ninhydrin	-	-	-	+++	+++	-	-	+	+++	-
Oxalates	Oxalate	++	-	++	-	+++	++	++	+++	-	-

A, acetone; H, hexane; M, methanol; D, decoction; I, infusion.

-, absent; +, weakly present; ++, present; +++, strongly present.

The presence of oxalates as a putative ANF and toxic factor in the extracts raised serious concerns in the study that compelled further investigations. Hence, a reversed phase HPLC method was adopted for the determination of oxalate in animal feed and *N. oleander*. The calibration curve was drawn with six standard solutions using a concentration range between 20 µg/mL and 100 µg/mL. The peak area was then plotted against the concentration. As a result, the regression equation was *y* = 15.051*x* + 127.45 with a correlation coefficient (*r*^2^ = 0.9968) that showed a good linearity.

**FIGURE 2:**
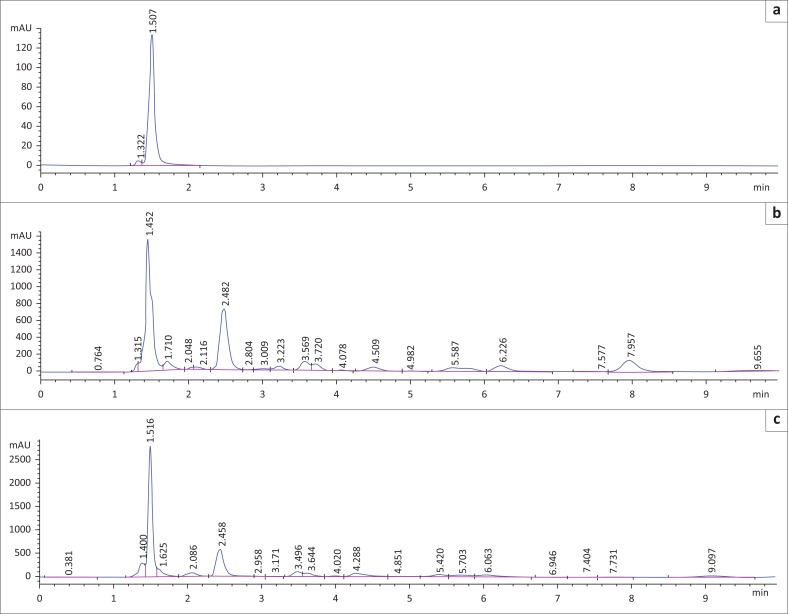
High-performance liquid chromatography chromatograms of analysed samples showing a common peak retention time at around 1.5 min: (a) sodium oxalate standard; (b) feed sample; (c) *Nerium oleander*.

The oxalate peaks in the samples were identified by comparing their retention times with that of the standard as shown in Figure 2. The oxalate peaks appeared at retention times of 1.515, 1.451 and 1.516 min for the oxalate standard, animal feed and *N. oleander*, respectively. The suspected oxalate peaks in the animal feed and *N. oleander* were confirmed by fortifications with oxalate standard. The oxalate contents and respective percentage yields in *N. oleander* and the animal feed were found to be 6.76 ± 0.245 mg/g (0.65%) and 5.74 ± 0.236 mg/g (0.55%), respectively (Table 2). The Mann–Whitney U test analysis confirmed that the results were statistically significant (*p* < 0.05).

**TABLE 2:** Oxalate contents (*n* = 5) and Mann–Whitney U test analysis.

Variable	Oxalate contents (mg/g per dry weight)		Calculated percentage per dry weight	
	Feeds	*Nerium oleander*	Feeds	*Nerium oleander*
**Replicates**				
	5.77	6.47	0.571	0.599
	5.70	6.81	0.553	0.668
	5.54	6.76	0.522	0.644
	6.12	6.63	0.572	0.620
	5.55	7.13	0.549	0.692
Average concentration (mg/g per dry weight)	5.74	6.76	0.553	0.645
Standard deviation	0.236	0.245	0.020	0.037

Mann–Whitney U test, *p* < 0.05.

A recovery test was performed using four replicates with 0.26, 0.33 and 0.39 mg of oxalate fortifications as shown in Table 3. In order to confirm the accuracy of the method, the average percentage recoveries were found to be within the acceptable range – between 80.3% and 98.5%. The calculated relative standard deviations (RSDs) for the recoveries were also within acceptable ranges.

**TABLE 3:** Recovery of oxalates in fortified *Nerium oleander* samples.

Mean amount of oxalates in samples (mg) (*n* = 4)	Mean amount of oxalate fortification (mg)	Determined oxalate contents (mg)	Oxalate recovered (mg)	Recovery (%)	RSD (%)
0.348	0.00	0.342	-	-	-
0.348	0.26	0.583	0.256	98.5	2.3
0.348	0.33	0.610	0.283	85.7	1.03
0.348	0.39	0.640	0.313	80.3	1.22

mg, milligrams; RSD, relative standard deviation.

Determination of precision based on intra-day showed a RSD of 0.99% and 1.13% at 0.33% and 0.39% fortification, respectively (Table 4), while percentage RSD of the repeatability and reproducibility were 2.58 and 2.21, respectively, as seen in Table 5. Moreover, the variance of measurement system for the two HPLC systems was found to be 0.037. The lowest fortification of the blank at a concentration of 5 parts per billion was used to determine the LOD and LOQ, which were found to be 2.38 *µ*g/mL and 7.22 *µ*g/mL, respectively.

**TABLE 4:** Determination of precision (intra-day).

Injected amount (mg)	Area	Oxalate determined (mg/mL)	Recovered (mg/mL)	Mean recovered (mg/mL)	Standard deviation	% RSD
0	5982	0.3890	-	-	-	-
	5050	0.3271	-	-	-	-
	4832	0.3126	-	-	-	-
	5159	0.3343	-	-	-	-
	5941	0.3862	-	-	-	-
	5082	0.3292	-	-	-	-
	4887	0.3162	-	-	-	-
	5128	0.3322	-	-	-	-
	5931	0.3856	-	-	-	-
	5184	0.3360	-	-	-	-
	4931	0.3192	-	-	-	-
	5130	0.3324	-	-	-	-
0.33	9249	0.6061	0.2795	0.2828	0.0028	0.9905
	9316	0.6105	0.2840	-	-	-
	9346	0.6125	0.2859	-	-	-
	9339	0.6120	0.2854	-	-	-
	9220	0.6041	0.2775	-	-	-
	9342	0.6122	0.2856	-	-	-
	9324	0.6110	0.2844	-	-	-
	9290	0.6088	0.2822	-	-	-
	9245	0.6058	0.2792	-	-	-
	9305	0.6097	0.2832	-	-	-
	9348	0.6126	0.2860	-	-	-
	9271	0.6075	0.2810	-	-	-
0.39	9731	0.6381	0.3115	0.3134	0.0036	1.134
	9841	0.6454	0.3188	-	-	-
	9725	0.6377	0.3111	-	-	-
	9718	0.6372	0.3107	-	-	-
	9725	0.6377	0.3111	-	-	-
	9857	0.6466	0.3200	-	-	-
	9700	0.6360	0.3095	-	-	-
	9763	0.6402	0.3136	-	-	-
	9762	0.6401	0.3136	-	-	-
	9821	0.6441	0.3175	-	-	-
	9708	0.6365	0.3200	-	-	-

mg, milligrams; RSD, relative standard deviation.

**TABLE 5:** Determination of repeatability and reproducibility.

High-performance liquid chromatography system	Peak area	Dilution factor	Total concentration (mg/g)	*x*	S^2^	S_r_^2^	S^2^*x*	S_R_^2^	S^2^_MS_	S_r_	S_R_	*x‾*	% RSD (S_r_^2^)	% RSD (S_R_^2^)
Agilent 1260	10 640.8	500	6.47	6.62	0.021	0.027	0.023	0.010	0.037	0.016	0.100	6.51	2.58	2.21
	10 807.4	500	6.76											
	10 804.6	500	6.63											
Agilent 1100	5951.10	400	6.49	6.40	0.033									
	4883.54	400	6.20											
	5139.17	400	6.53											

mg, milligrams; S^2^, sample variance; *x*, sample mean; S_r_^2^, repeatability; S^2^*x*, variance of *x*; S_R_^2^, reproducibility; S^2^_MS_, variance of measurement system; S_r_, standard deviation of repeatability; S_R_, standard deviation of reproducibility; x‾, average of *x*; RSD, relative standard deviation.

## Discussion

**FIGURE 3:**
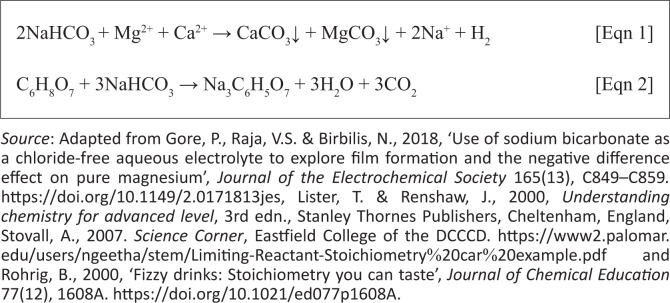
Chemical reactions that occurred in the infusion. Equation 1 (Gore, Raja & Birbilis 2018; Lister & Renshaw 2000), precipitation of calcium and magnesium carbonates; Equation 2 (Rohrig 2000; Stovall 2007), Formation of sodium citrate that sequestrates Ca^2+^ and Mg^2+^ ions.

The objective of the study was to investigate the effects of organic and aqueous extraction techniques on animal feed and plant leaves, as well as to quantify oxalates as putative antinutritive and toxic factors. This was based on the observation that currently there is no single and effective method used to remove a wide range of these factors (Akande & Fabiyi 2010). Removal of these factors remains a major challenge because a combination of two or more methods is generally required. This particularly affects farmers in developing African countries with poor infrastructures and limited resources. Two extraction techniques were evaluated and higher extraction yields were obtained in the infusions as expected because of the effects of water softness. According to the WHO (2011), the presence of the cations, especially divalent ones such as Ca^2+^ and Mg^2+^, in the decoction were engaged in the formation of insoluble mineral deposits, which play a major role in reducing the efficiency of water. These cations were therefore precipitated in the presence of sodium hydrogen carbonate as shown in Equation 1 (Figure 3). To avoid further complications during extraction, the precipitated carbonates were dissolved in the presence of citric acid (Equation 2). The resulting sodium citrate (Na_3_C_6_H_5_O_7_) inactivated water hardness by sequestration or chelation of the Ca^2+^ and Mg^2+^ ions to form water-soluble complexes (Harding 1991).

Alternatively, aqueous extractions proved to be safe, reliable and affordable methods as traces of undesirable components such as organic solvents were not detected in the resulting animal feed or foliage. However, non-detection of the phytochemicals in the extracts does not necessarily mean their absence in the raw animal feed or foliage, but it does address the capacity of the extraction technique employed. A boiling process (decoction) of the *N. oleander* was shown to reduce several factors including cardiac glycosides, saponin glycosides, flavonoids, tannins, alkaloids, anthraquinones and amino acids, which is comparable with previous findings (Ndidi et al. 2014). Even though the infusion was unable to reduce the flavonoids and tannins, it effectively reduced factors, including the terpenoids, phenols and oxalates.

Oxalate contents of 3.2% and above were reported to cause acute toxicity in livestock (Cymbaluk, Miller & Christensen 1986). In contrast, in this study, the levels of oxalates found in both animal feed and *N. oleander* were relatively low and thus deemed safe, as they were within the expected range of 0.14% – 0.83%.

### Practical implications

Trees and shrubs contain poisonous compounds and free browsing of livestock on these plants poses a serious threat to farmers. Hence, studies that analyse the antinutritive and toxic factors in trees and shrubs to which livestock might be inadvertently exposed, could possibly lead to solutions that prevent loss of livestock. The current study investigated the effects of extraction techniques on which farmers may rely to remove the undesirable toxic factors that may lead to livestock poisoning when giving foliage as supplements. It also contributed in the development of the methods for quantification of the oxalates, which may be applied to quantify oxalates extracted from other biological systems.

### Limitations of the study

There are many different antinutritive and toxic factors found in plants and feeds, and the study focused mainly on the selected factors, such as cardiac glycosides, saponin glycosides, sterols, terpenoids, anthraquinones, phenols, alkaloids, tannins, oxalates, flavonoids, proteins and amino acids. Freshly collected plant material is frequently used immediately to avoid unnecessary physiological changes before extraction (Odebiyi & Sofowora 1978). However, the material used in this study was dried, as only dry feeds were available from the Agricultural Research Council stores.

There are several different tests available for testing phytochemicals, for instance, cardiac glycosides. In this study, we focused on the Lieberman and Salkowski tests. Future studies will test the extracts produced in this study using other available methods, and comparisons will be made.

## Recommendations

A great deal of antinutritive and toxic factors from various plant foliage can be removed using the aqueous extraction technique, with the benefits that it is cheap, simple and safe to use. However, both the extract and the resulting feed or foliage should be analysed for the presence of these factors to account for their overall contents. Subsequent to the qualitative analysis, confirmatory tests to rule out false-positive reactions should be carried out with all extracts that show positive results. In addition, the developed method for the oxalates can be used across a wide range of plant species or any other species with a different matrix.

## Conclusion

It is necessary to develop methods for removal of undesirable antinutritive and toxic factors in feeds and foliage in order to reduce the negative impact that they potentially pose to livestock. The present work confirmed the efficacy of the extraction techniques explored and analysis of these factors in *N. oleander* and one source of animal feed. Of the two extraction techniques explored, aqueous extraction proved to be more valuable to use relative to the organic extraction technique in terms of safety, reliability and efficiency. Water hardness was one of the factors with major implications on extraction efficiencies. Therefore, soft water was used for the infusion, as it produced the best aqueous extraction method that does not require boiling, which may eventually leave traces of degraded poisonous compound residues in animal feeds or foliage.

Additionally, the developed HPLC method was accurate, precise, repeatable and reproducible for the determination of the oxalates. Although the levels of oxalate contents were relatively low, freely browsed plants may pose a serious threat to livestock health; hence removal of antinutritive and toxic factors is highly recommended. The method described in this study can be used to determine the levels of free oxalic acids as they exist in tree or shrub foliage and to study the oxalate synthesis under different stress conditions.
